# Gamified Optimized Diabetes Management With Artificial Intelligence–Powered Rural Telehealth Intervention (GODART): Protocol for an Optimization Pilot and Feasibility Trial

**DOI:** 10.2196/70271

**Published:** 2025-12-05

**Authors:** Tapan Mehta, Tejossy John, Aseel El Zein, Victoria Faught, Tanjila Nawshin, Tejaswini Subhash Chilke, Caroline W Cohen, Andrea Cherrington, Mohanraj Thirumalai

**Affiliations:** 1 Department of Family and Community Medicine Heersink School of Medicine University of Alabama at Birmingham Birmingham, AL United States; 2 Department of Health Services Administration School of Health Professions University of Alabama at Birmingham Birmingham, AL United States; 3 Lakeshore Research Collaborative School of Health Professions University of Alabama at Birmingham Birmingham, AL United States; 4 Division of General Internal Medicine and Population Health Heersink School of Medicine University of Alabama at Birmingham Birmingham, AL United States

**Keywords:** telehealth, type 2 diabetes, lifestyle modification, digital health, artificial intelligence

## Abstract

**Background:**

Type 2 diabetes mellitus (T2DM) is highly prevalent in the United States and represents a significant public health challenge. Telehealth interventions have shown promise for improving T2DM outcomes, but their effectiveness is often limited by disparities in digital literacy and access, especially in rural areas. To address this gap, we propose an innovative, individualized lifestyle modification intervention delivered via phone call to support glycemic control.

**Objective:**

This paper outlines the protocol for a pilot study designed to assess the feasibility and preliminary effectiveness of an artificial intelligence–assisted intervention for T2DM self-management in rural settings.

**Methods:**

The study uses the preparation phase of the MOST (Multiphase Optimization Strategy) framework to evaluate two components: (1) automated versus human health coaching and (2) fixed versus adapted gamified financial incentives, based on participants’ daily engagement with automated monitoring calls. We aim to enroll 88 adults with T2DM and hemoglobin A_1c_ (HbA_1c_) levels between 6.5% and 11.5%. Participants receive daily interactive voice response calls tracking diet, physical activity, medication adherence, and blood glucose, and weekly coaching based on randomization. In the fixed-reward arm, participants earn US $0.60 per day; in the adaptive arm, rewards start at US $0.20 and increase weekly, with penalties for missed days. Primary outcomes include feasibility metrics and preliminary changes in HbA_1c_. Semistructured interviews will assess patient experience.

**Results:**

This study was funded by the National Institute of Diabetes and Digestive and Kidney Diseases. As of October 2025, we have enrolled and completed data collection for 88 participants. We expect to complete the feasibility analysis by December 2025.

**Conclusions:**

This pilot and feasibility study evaluates a low-tech, artificial intelligence–assisted T2DM intervention designed to reduce digital barriers and inform a future MOST optimization trial.

**Trial Registration:**

ClinicalTrials.gov NCT05344859; https://clinicaltrials.gov/study/NCT05344859

**International Registered Report Identifier (IRRID):**

DERR1-10.2196/70271

## Introduction

Type 2 diabetes mellitus (T2DM) is a major public health crisis exerting a considerable burden on individual health and the health care system across the United States. Its prevalence is especially high in the Deep South of the United States and in rural areas compared to urban settings [1-3]. Glycemic control through a combination of diet, physical activity (PA), glucose monitoring, and medication adherence has been shown to be effective in T2DM management (reduced hemoglobin A_1c_ [HbA_1c_]) [4-8]. However, the majority of individuals with T2DM are unable to adhere to the diabetes management guidelines, and this problem is amplified in populations with low health literacy, higher poverty levels, or in remote rural settings [9-11].

T2DM significantly impacts clinical resource utilization because of its frequent comorbidity with cardiovascular and chronic kidney diseases [12-14]. Primary care providers serve as the initial point of contact for most patients with chronic conditions [15] and handle, on average, 4.6 problems per patient visit for patients with diabetes [14,16]. This significant workload and the shortage of primary care professionals lead to primary care access issues, highlighting the need to empower patients’ behavior modification outside of the clinic. Increasing patients’ knowledge and confidence to self-monitor and modify their health behavior is crucial to improving their health outcomes while conserving limited clinic resources.

Regular self-monitoring (tracking) helps informed decision-making [17-19]. For example, regular blood glucose monitoring provides objective feedback on glycemic control [20]. Similarly, tracking PA (steps) and dietary intake helps alter respective behaviors [21,22] and can influence medication adherence behaviors [23]. Therefore, self-monitoring (tracking) of behaviors has great potential to affect diabetes related health behaviors. However, maintaining diaries or apps for diabetes self-management activities has been shown to increase participant burden and reduce long-term engagement due to loss of interest, complexity, and digital literacy barriers [24,25]. While self-monitoring alone sees benefits, this intervention is often paired with health coaching to provide direct feedback on behaviors, plus provide goal setting and suggestions of dietary or PA alternatives where appropriate. Indeed, digital health coaching has been shown to be effective in improving T2DM outcomes, particularly lowering HbA_1c_. A systematic review and meta-analysis of 28 studies, 82% of which were randomized trials, found that high-intensity digital coaching interventions are more effective in managing T2DM when compared to usual care [26].

Despite self-monitoring and health coaching’s potential to aid in diabetes self-management, the effectiveness of their delivery is hindered by the digital divide [27]. The adoption of digital technologies like smartphones and tablets involved in such interventions is growing, but not uniformly, and sees disparities by age, gender, education, and income. For example, while 97% of adults aged 30-49 years have a smartphone, this rate drops to 91% for adults aged 50-64 years and to only 79% of adults aged 65 years and older [28,29]. Even when given access to a tablet or smart device, many adults are not sufficiently technically proficient to operate them or the software or apps loaded onto them [30]. To ensure broader accessibility and maintain digital equity, there is a need for a scalable, minimally digital intervention that can operate without a mobile app or high-speed internet connectivity. In response to this, the proposed study’s intervention leverages the most pervasive and simplest technology—a telephone call, with no expectations of internet availability or smart device-based application proficiency. Use of telephone calls ensures inclusiveness and minimizes barriers for those who may lack or be unable to use smartphones or texting features for either self-monitoring or health coaching.

A telephone call-based self-monitoring and health coaching intervention would be responsive to the need for tech-agnostic self-monitoring for T2DM, but it is human resource-heavy. Hence, we are proposing artificial intelligence (AI)–assisted daily monitoring and automated health coaching as a scalable and low-cost alternative using the interactive voice response (IVR) system. The goal is to use accessible technology to optimize the reach and effectiveness of health coaching. To date, no study has reported using telephone calls for AI-assisted daily monitoring and health coaching focused on the four essential behaviors for glycemic control—PA, diet, medication adherence, and blood glucose monitoring. The daily nature of these automated calls for behavior monitoring presents a challenge for adherence. Rewards, especially monetary rewards, have been shown to provide significant extrinsic motivation toward behavior changes [31-33]. Gamification is also increasingly recognized as an effective method to stimulate adherence [34,35]. Very few studies have compared adaptive gamified financial incentives with fixed financial rewards. We incorporate gamification by implementing a gamified rewards scheme [31] based on participants’ consistency of answering daily self-monitoring calls to improve self-monitoring (tracking) behavior. GODART’s (Gamified Optimized Diabetes Management with Artificial Intelligence-Powered Rural Telehealth) theoretical framework is based on the social cognitive theory (SCT) for behavior change.

The proposed study aims to pilot GODART’s core feature: an automated behavior monitoring program using natural language processing (NLP) and two intervention components. The first intervention component (technology optimization experiment) will assess the feasibility of automated coaching and human coaching, and estimate preliminary effects of automated coaching compared to human coaching. The second intervention component (resource optimization experiment) will assess the feasibility of fixed and adaptive gamified rewards and estimate the preliminary effects of adaptive rewards compared to gamified rewards. Overall, we will assess the feasibility of our GODART study protocol in terms of recruitment, retention, and adherence. We hypothesize that the proposed interventions will be feasible and acceptable, and show early promise for reducing HbA_1c_ in adults with T2DM.

## Methods

### Overview

**Figure 1:**
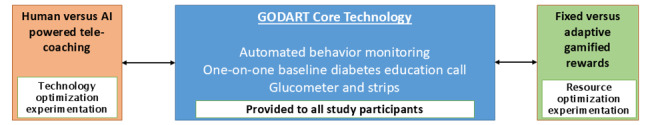
Overview of the GODART intervention structure; all study participants receive the GODART core technology (ABMS), but are randomized to one of two optimization experimental conditions: the Technology Optimization Experimentation, comparing human versus automated health coaching, or the Resource Optimization Experimentation, comparing fixed versus adaptive gamified rewards. ABMS: automated behavior monitoring system; AI: artificial intelligence; GODART: Gamified Optimized Diabetes Management with Artificial Intelligence-Powered Rural Telehealth.

For GODART, we developed an automated behavior monitoring system (ABMS) where participants receive phone calls to log their exercise, medication, and diet behavior each day for 24 weeks. Calls are delivered through IVR and transcribed using AI features available in Twilio software (Twilio Inc), removing the need for a human operator and allowing participants to report their daily behavior through natural speech. Rewards for completing calls are issued either at a flat rate or at an adaptive (gamified) rate. ABMS calls are supplemented by either human or automated IVR-delivered health coaching once per week. GODART’s theoretical framework, based on the SCT for behavior change, is outlined in Textbox 1, and an overview of GODART’s intervention structure is detailed in Figure 1.

**Social cognitive theory framework application in GODART (Gamified Optimized Diabetes Management with Artificial Intelligence-Powered Rural Telehealth).**

**Outcome expectations**

- Definition: Anticipated outcomes to behavior change (diet, physical activity, medication adherence, and daily blood glucose monitoring)
- Change strategy: Coaching calls (both human and automated) will address any negative-outcome expectations. The educational content will have content focused on outcome expectations.

**Social support**

- Definition: Support from family and friends for participation in modified behaviors.
- Change strategy: All educational content and coaching calls will stress the importance of these behavior changes, irrespective of one’s diabetes status. Thus, we will recommend including friends and family in this journey of transforming behaviors.

**Self-efficacy**

- Definition: Confidence in one’s ability to exhibit positive behavior in specific situations.
- Change strategy: Our coaching call will, on a weekly basis, choose different specific situations and focus on positive behaviors during those situations. Our behavior-monitoring calls will set small incremental (adapted) goals, leading to increased self-efficacy. Coaching calls will include relevant content.

**Self-regulation**

- Definition: Exerting self-control over behavior.
- Change strategy: First, the core of our project is centered on behavior monitoring and providing feedback, which aids self-regulation. The fixed versus adapted rewards proposed are aimed at providing further extrinsic motivation toward self-regulation. Finally, the coaching calls will focus on stressing the importance of self-regulation.

### Ethical Considerations

This study was approved as expedited by the University of Alabama at Birmingham (UAB) Institutional Review Board under record IRB-300008752 and registered with ClinicalTrials.gov (NCT05344859). Informed consent was obtained for all participants either electronically using REDCap (Research Electronic Data Capture; Vanderbilt University) or, if needed, through telephone or a mailed physical copy. Participant data are deidentified to maintain privacy and confidentiality. Participants were compensated up to US $225.80 for their participation in study monitoring calls and attending study visits.

### Study Design

**Figure 2:**
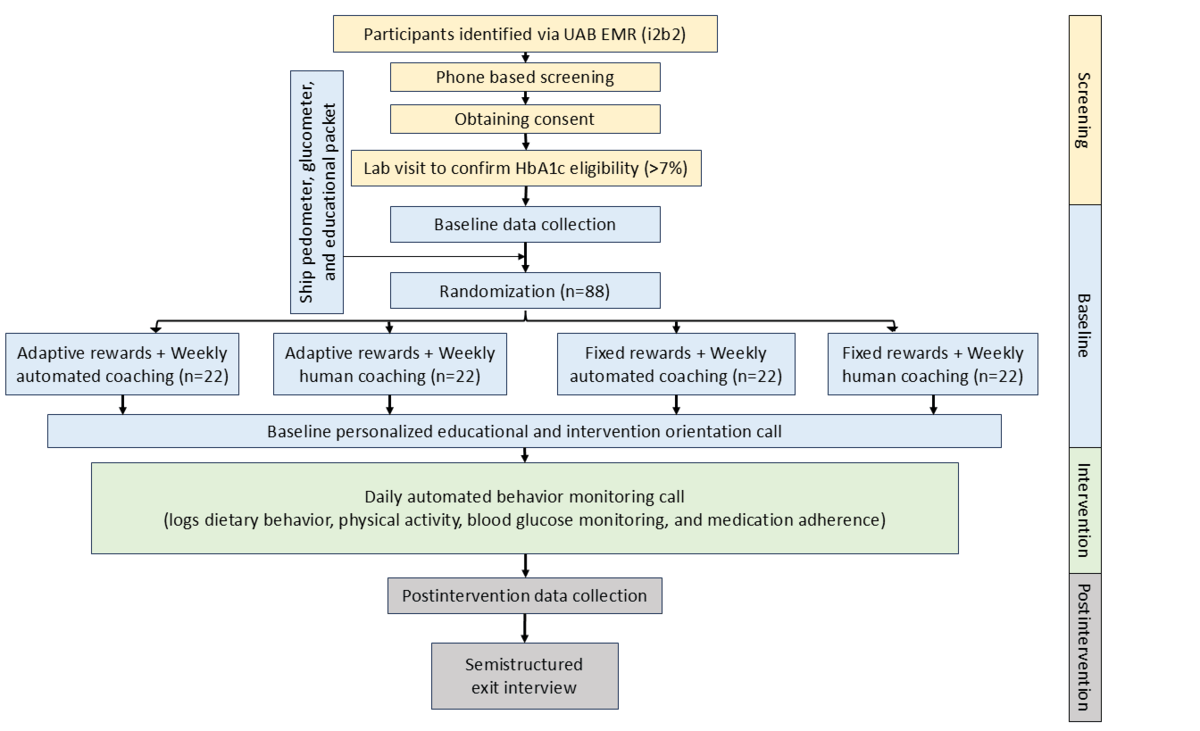
Flow diagram outlining screening, randomization (n=88), and intervention arms comparing fixed versus adaptive incentives and automated versus human coaching in the GODART study. EMR: electronic medical record; GODART: Gamified Optimized Diabetes Management with Artificial Intelligence-Powered Rural Telehealth; UAB: University of Alabama at Birmingham.

The GODART study uses the MOST (Multiphase Optimization Strategy) design, grounded in resource management principles and continuous improvement [36-38]. This study aligns with the preparatory phase of MOST. We are applying factorial experimentation as part of this MOST preparatory phase to assess the feasibility of two different intervention components (experimentations), with two levels in each group, yielding four experimental conditions. The Technology Experimentation component (Figure 1) focuses on comparing automated coaching through IVR with traditional human-delivered coaching. The goal here is to assess the feasibility of optimizing health coaching by leveraging accessible technology and assessing the preliminary effect when compared to human coaching. The Resource Experimentation focuses on assessing the feasibility and preliminary effect of the adaptive (gamified) rewards when compared to the fixed rewards program. Upon completion of the intervention, all participants will be invited to complete an exit interview to gauge expectations and perceived benefits and to collect suggestions for protocol improvement. Study findings will help us assess the feasibility of the protocol, acceptability of the intervention components, and their preliminary effectiveness in reducing HbA_1c_, leading to a confirmatory optimization trial. Figure 2 shows a schematic diagram of the study flow.

### Participants and Eligibility Criteria

This study aimed to recruit a total of 88 patients diagnosed with T2DM [39]. Eligible participants must be individuals aged 18 years or older, can speak English, and have HbA_1c_ between 6.5% and 11.5%. English language proficiency was included as an eligibility criterion, as the telephone-based intervention is delivered in English and requires a level of comprehension sufficient for participants to understand the instructions and interact meaningfully with the system. Additionally, due to resource constraints and the pilot nature of this study, we limited recruitment to English-speaking individuals to ensure feasibility and optimize initial implementation. Participants with HbA_1c_ within the range of 6.5% to 11.5% were included in the study to ensure that we target individuals who will benefit from improved self-management and behavior modification.

Exclusion criteria include current enrollment in other diabetes and weight management interventions, speech or hearing impairments, a major cardiac event in the 6 months prior to screening, current or imminent pregnancy, and ongoing use of insulin. Additionally, participants are excluded if they are unwilling to accept random assignment, engage in regular intervention-related phone calls, or complete study procedures. Individuals currently enrolled in other diabetes or weight management interventions are excluded to prevent confounding effects and to isolate the impact of the study interventions. As the intervention relies on telephone-based communication, individuals with hearing or speech impairments are not included. Participants with major cardiac events, ongoing insulin therapy, or current or imminent pregnancy are also excluded due to safety concerns and the need for more intensive or specialized diabetes management.

### Recruitment and Retention

Participants were identified and recruited using the National Institute of Health–funded Integrating Biology and the Bedside (i2b2) system implemented at the UAB Health System. This self-service tool allows users to build a cohort of patients by querying clinical data and developing a recruitment database for the study. Recruitment occurred from patients across the health system, which included community clinics that serve patients with Rural-Urban Commuting Area codes of ≥4, which includes all nonmetropolitan tracts, specifically micropolitan, small towns, and isolated rural areas (ie, large towns or clusters) [40]. Using the recruitment database, opt-out letters [41] were sent to all residences of potentially eligible patients either via mail or email. The opt-out letter informed individuals about the study and allowed them to decline contact regarding the study by calling or sending an SMS text message to the phone number provided. Only those who did not opt out within 2 weeks were contacted regarding their interest in participating in the study. Additional recruitment strategies included posting study flyers in high-traffic and clinical areas among UAB clinics and collaborating with clinical staff and providers to identify and refer eligible patients. We also sent the study referral to an application that is integrated within the electronic health record system. The application nudges primary care providers about the study eligibility when a potentially eligible patient visit is scheduled.

Our retention efforts include a combination of strategies to ensure participants remain engaged throughout the study. Participants receive regular reminders and check-in phone calls, and study visits are conducted at LabCorp locations close to their residences to reduce the travel burden. The interventions are delivered remotely, minimizing the need for on-site treatments, and participants receive monetary compensation as part of the participation rewards. Additionally, they provide alternative contact information to ensure consistent communication. All participants receive IVR calls throughout the study as part of the engagement strategy. If any participant stops responding, the research coordinator contacts them directly to address any issues. Participants are contacted per the approved Human Subject Protocol. Up to 3 attempts are made to reach out to each participant via phone call, voicemail, or text to verify whether missed calls were due to technical issues or an intention to withdraw from the study. If a participant fails to respond to three consecutive contact attempts, no further outreach is conducted, and the participant is classified as lost to follow-up and removed from the calling list.

### Screening

All patients first undergo telephone-based screening to confirm eligibility. For electronic consent, participants receive a consent form via email using REDCap, a secure web app for managing research data. If the participant is unable to complete the consent form electronically, either a phone consent is obtained or a physical copy of the consent form is sent to the participant’s mailing address. A completed, signed consent form is then uploaded to REDCap, and physical copies are stored in secure cabinets at the research site. Each participant is asked to visit a LabCorp location closest to the patients’ home addresses to provide a fasting blood sample to be analyzed for HbA_1c_ and lipid profile (ie, low-density lipoprotein cholesterol, high density lipoprotein cholesterol, very low density lipoprotein, triglycerides, and total cholesterol) and biometrics (ie, blood pressure, height, weight, and waist circumference). Measurements are recorded to the nearest 0.1 inches (in) for height and waist circumference and to the nearest 0.1 pounds (lb) for weight.

### Baseline Assessments

Once participant eligibility is confirmed from screening, the blood values used in the in-person screening visit described above are used as baseline values. In addition, a baseline survey packet consisting of sociodemographic questions and five validated survey instruments is sent electronically to the participants. The survey instruments are as follows: The Spoken Knowledge in Low Literacy in Diabetes Scale (SKILLD) assesses patients’ understanding of diabetes management [42]; the Patient Health Questionnaire-4 screens for symptoms of anxiety and depression [43]; The Diabetes Distress Scale measures the emotional burden associated with diabetes management [44]; The Diabetes Empowerment Scale evaluates patients’ self-efficacy in controlling diabetes [45], and the EQ-5D [46] survey assesses health-related quality of life. All study data are collected and securely stored in the REDCap database. If participants are unfamiliar or uncomfortable with completing the survey packet electronically or if they lack an email address, the survey packets are mailed to their preferred address. Included in the packet is a prepaid stamped envelope to return the survey packet. Upon receipt, survey responses are uploaded to REDCap, and the physical copies are stored in a secure cabinet at the research site.

### Delivery of the Pedometer, Glucometer, and Educational Packet

Before the intervention began, participants across all arms of the study were shipped a pedometer, a glucometer with strips for 6 months, and a T2DM educational packet. The education content is highly pictorial and is based on the Diabetes Literacy and Numeracy Education Toolkit [47], a packet designed with low-literacy principles and visual aids, for T2DM education and management in patients with low literacy and numeracy skills. In addition, educational patient handouts from the Centers for Disease Control and Prevention (the National Diabetes Education Program and Diabetes Prevention Program) corresponding to all content areas measured by the SKILLD were included in the packet [48]. Topics covered in these materials included special considerations for exercise, understanding HbA_1c_, eye health, foot exams for individuals with T2DM, and visual aids for making healthy food replacements to manage blood sugar. A guide for estimating portion sizes was also included to facilitate accuracy with daily diet tracking. Sending the educational packet ensures that all participants have access to the same basic principles of diabetes management before starting the intervention.

### Randomization

Computer-generated randomization assignment was done for all consented participants. We implemented a permuted block randomization with varying block sizes, blinded to the assessment team. Participants were randomized to four experimental conditions based on a 2 by 2 factorial design with equal probability allocation. The randomization list is concealed within the REDCap randomization module. We have programmed this such that the participant can be randomized and allocated only to an experimental condition after it is confirmed that baseline data have been collected and entered. Once the primary outcome measures are entered in REDCap, the study staff members can assign the participants to their respective experimental conditions. The statistician and the Principal Investigators are blinded, but the participants and the health coach are not blinded. All participants enrolled in the study were randomized to one of the four arms:

- Arm 1: Adaptive reward+weekly automated coaching
- Arm 2: Adaptive reward+weekly human coaching
- Arm 3: Fixed reward+weekly automated coaching
- Arm 4: Fixed reward+weekly human coaching

### Initial Personalized Orientation Session

All participants received an initial orientation session. Study team members served as health coaches, including a clinical registered dietitian and a certified health coach. During this session, the team reviewed the SKILLD survey completed as part of the baseline survey to identify gaps in the participants’ knowledge of T2DM management. These gaps were then addressed, and an introduction to the intervention was provided. The Diabetes Literacy and Numeracy Education Toolkit educational packet was used by health coaches during the orientation process to ensure all participants have equivalent baseline knowledge regardless of their literacy level. For participants with limited literacy skills, the health coaches used this call to walk participants through the educational materials, focusing on the visual components and providing detailed verbal explanations of all key information. This individualized approach ensured that participants who cannot read are still able to access, understand, and engage with the educational content, thereby establishing a consistent foundation of T2DM management knowledge across the study population.

This call provided key information about the intervention, including expectations for the telephone-based ABMS and health coaching calls, participants’ randomization to fixed or adaptive rewards, and recommended use of a glucometer and pedometer. Participants also listened to a sample ABMS call to learn best practices for voice recognition when reporting step counts, medication use, and blood glucose testing.

The call included instructions to improve the completeness and accuracy of diet records, with reminders about commonly forgotten items (eg, beverages, condiments, and sweeteners) and guidance to avoid brand names for better matching in Nutritionix. Participants also received introductory nutrition education focused on making healthy replacements to support glycemic control, specifically limiting added sugars, refined carbohydrates, and saturated fats. These behaviors are tracked through daily ABMS calls and discussed during weekly health coaching sessions to enhance both the accuracy and efficiency of self-monitoring.

Following the orientation call, all participants were activated in the ABMS and began receiving calls starting the following Monday.

### Intervention Components

#### Automated Behavioral Monitoring System

All participants receive daily calls through the ABMS using IVR technology for a 24-week period. Each participant selects a preferred call time during their initial orientation session with a health coach. If a call is missed, the system automatically places a reminder call 30 minutes later. Participants who miss both calls can return the call at their convenience to record their data.

**Figure 3:**
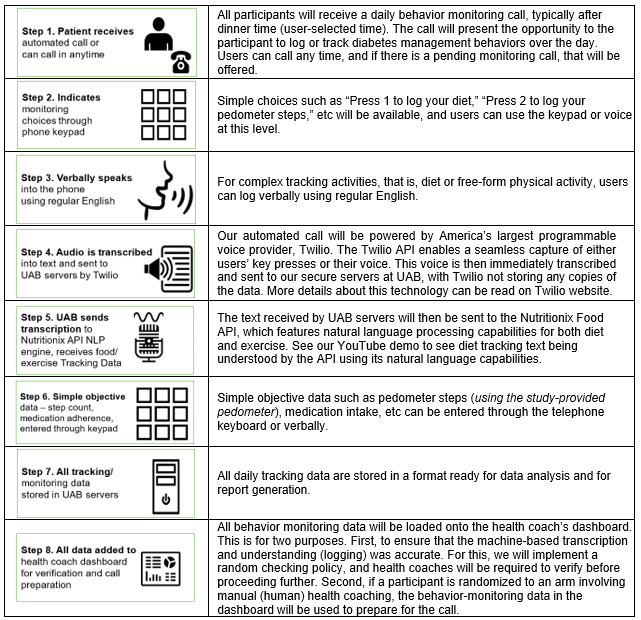
Overview of the automated behavior monitoring system: 8-step process used in the GODART study to support diabetes self-management. See our YouTube demo [49] for step 5. API: application programming interface; GODART: Gamified Optimized Diabetes Management with Artificial Intelligence-Powered Rural Telehealth; NLP: natural language processing; UAB: University of Alabama at Birmingham.

Unlike traditional self-monitoring approaches that rely on written logs or mobile apps, the ABMS enables participants to speak naturally during calls—for example, “For breakfast, I had a muffin, a banana, and a cup of coffee” or “Today I walked about 5000 steps.” The system captures and processes this information seamlessly using AI-assisted NLP and nutrition databases. The ABMS is designed to collect daily data on four key self-management behaviors: dietary intake, PA, medication adherence, and fasting blood glucose monitoring (yes or no). Figure 3 outlines the 8-step structure of the ABMS used to support diabetes self-management in this study.

During the daily IVR calls, participants report their PA (eg, pedometer step count and engagement in moderate or vigorous activity), medication adherence (eg, whether medications were taken, taken as prescribed, and at the correct time), fasting blood glucose monitoring (yes or no), and dietary intake (meal-specific foods and beverages, including quantities). Textbox 2 lists the daily monitoring questions delivered by the IVR system, and the full branching logic is detailed in Multimedia Appendix 1.

**ABMS (automated behavior monitoring system) daily monitoring call questions.**

Category and sample questions

**Daily PA (physical activity)**

- Have you experienced any physical symptoms or complaints that could interfere with your PA?
- Did you wear your Fitbit or pedometer today? If yes, how many steps did you take today?
- Did you participate in any moderate intensity or greater PA today? If yes, approximately how many minutes did you spend participating in moderate intensity or greater PA today?

**Daily medication adherence**

- Did you take your diabetes medications today? If yes, did you take your diabetes medications in the amount prescribed by your doctor? If yes, did you take your diabetes medications at the appropriate time today?

**Daily blood sugar monitoring**

- Did you test your blood sugar in the morning today?

**Daily food intake**

- Did you have breakfast today? If yes, what did you eat or drink for breakfast? [Participant instructed to provide the amount of each food or drink]
- Did you have lunch today? If yes, what did you eat or drink for lunch?
- Did you have dinner today? What did you eat or drink for dinner?
- Was there anything else you had today? This could be a snack or a drink.

Our IVR-based ABMS is programmed to interact with both key presses and voice commands [50,51]. Keypresses are used for binary (yes or no) questions and numerical data, such as step counts, while dietary intake is captured exclusively through voice input. The system is built using a MySQL database and Laravel, a PHP-based framework. For telephony, the ABMS uses Twilio’s application programming interface (API), which allows the system to immediately retrieve participant inputs, including keypress responses and transcribed voice recordings.

Yes or no and numerical responses are directly stored in the MySQL database. For dietary data, transcribed voice inputs are processed in real time using the Nutritionix API (Nutritionix, Bethesda, MD)—a commercial nutrition database that supports NLP. Nutritionix has been widely used as an NLP nutrition tracking system in prior research [52,53]. All calls are recorded, and the raw audio recordings, transcriptions, and nutrition outputs from the API are stored and made available to study staff for quality checks, such as identifying discrepancies or troubleshooting system limitations. All ABMS calls follow the same structure and are delivered uniformly to all study participants.

There are two experimental groups (two levels in each), which are explained below.

#### Experimentation Group 1: Automated Versus Human Health Coaching

Participants receive weekly human health coaching or automated health coaching for 24 weeks. Participants randomized to human health coaching receive a call from a human health coach, while those randomized to automated health coaching receive a rules-based automated health coaching call delivered via IVR. Topics include meal timing and spacing, monitoring nutrients for glycemic control, benefits of regular exercise, medication adherence, and consistent blood glucose monitoring.

Both coaching methods rely on data from the prior week’s AI-assisted ABMS calls, which are populated into a coaching dashboard using Nutritionix and participant-reported data. In both coaching methods, the human health coach reviews participants’ intake and performance against weekly goals. However, the scope and delivery of feedback differ. In the automated health coaching method, participants receive feedback via an IVR system using a rule-based AI engine. The human health coach provides weekly dietary suggestions specifically related to carbohydrate and saturated fat intake. The system flags whether the intake in these categories is within range or elevated, and highlights contributing food items. Based on this information, the coach inputs tailored suggestions that are aligned with participants’ health goals, which are then delivered automatically via IVR. Feedback related to medication adherence, blood glucose monitoring, and PA is also delivered automatically via IVR, based on participant-reported data. In the human health coaching method, the same intake data are reviewed, but coaching occurs through direct human interaction, allowing for more nuanced discussion around dietary patterns, behavior change strategies, and personal barriers. The coach is able to engage participants in deeper conversation, explore the context behind food choices, and collaboratively problem-solve to support goal attainment.

#### Experimentation Group 2: Adapted Versus Fixed Gamified Reward Levels

Participants in this group are randomized into two arms: fixed-reward levels and adapted gamified reward levels; the total possible amount earned over the period of 24 weeks in each arm is US $100.80. The purpose of the rewards is to conduct daily self-monitoring, not as incentives per se to participate in the study. In the fixed-reward arm, participants receive 60 cents per day for answering daily monitoring calls for 24 weeks, earning up to US $100.80 if they answer daily. In the adapted gamified reward arm, participants’ daily rewards increase weekly, starting at US $0.20 per day in the first week, US $0.25 per day in the second, US $0.30 per day in the third, and US $0.65 per day from the fourth week until the end of the study at 6 months. In this arm, missing one day of monitoring (in the past 7 days) drops the reward value by one level (eg, from US $0.65 to US $0.30), with additional missed days dropping the reward further. Participants can rebuild their reward levels by consistently answering daily calls. Like the fixed award arm, a participant answering the call daily for 24 weeks receives up to US $100.80. This resource management–driven study arm will inform us of the extent to which rewards can enable successful behavior change.

### Postintervention Assessments at Month 6

At month 6, similar to the baseline visit, each participant is asked to visit their nearest LabCorp location to provide a blood sample for HbA_1c_, lipid profile, and biometrics. Participants are also sent a 6-month survey packet either electronically via email using REDCap or via physical mail, depending on the participant’s preference. This packet includes all of the surveys in the baseline packet in addition to: Speech User Interface Service Quality, which evaluates the quality, perceived ease of use, and user satisfaction of speech-based interfaces [54]; System Usability Scale, which measures system usability [55]; and the theoretical framework of acceptability survey [56] which assesses the acceptability of the intervention.

### Semistructured Exit Interview

Upon completion of the intervention at month 6, participants are invited to complete a semistructured exit interview conducted over a phone call and recorded, to gauge expectations, perceived benefits, and suggestions for protocol improvement (see Multimedia Appendix 2 for interview guide).

### Treatment Fidelity

Monitoring fidelity is essential to ensuring the intervention’s internal and external validity. In that context, we have developed a fidelity-monitoring plan (Table 1) to address the five areas of intervention fidelity outlined by the National Institute of Health Behavior Change Consortium: design of study, provider training, delivery of treatment, receipt of treatment, and enactment of treatment. Monitoring fidelity is done by the study coordinator, along with the support of the programmer, who provides periodic reports.

**Table 1:** Overview of fidelity measures and areas addressed.

Data source	Monitoring frequency	Study design	Coach training	Intervention delivery	Intervention receipt	Intervention enactment
Coaching call checklist	Monthly	✓	✓	✓		
Coaching call logs	Monthly	✓		✓		
Content resource bank	Quarterly	✓				
Review of the dashboard and its event log	Weekly	✓		✓		
Review of participant food, physical activity, and medication	Weekly				✓	✓
Team meetings to discuss participant progress and protocol adherence	Biweekly	✓	✓	✓	✓	✓

### Outcomes

We are collecting feasibility, process, clinical, and patient-reported outcomes (PROs) during the intervention, with feasibility outcomes serving as the primary end points. As outlined in Tables 2 and 3, our primary outcomes include key feasibility indicators: recruitment, retention, and adherence to the intervention. Secondary outcomes include other feasibility measures such as process measures (eg, time to recruit, percentage of missing data, and enrollment rate), and selected patient-centered and clinical outcomes. The choice of clinical and patient-reported assessments is based on guidelines from the American Diabetes Association [57]. Specifically, diabetes distress is a key PRO, while HbA_1c_ and weight are the key secondary clinical outcomes. Exploratory outcomes include additional PROs (eg, quality of life, depression, self-efficacy, and acceptability of the intervention) and clinical indicators such as blood pressure and lipid profile. PROs are collected using validated instruments such as the Diabetes Distress Scale, EQ-5D, Patient Health Questionnaire-4, and the Diabetes Empowerment Scale. Intervention acceptability is assessed using the Speech User Interface Service Quality, System Usability Scale, and theoretical framework of acceptability scales, in addition to qualitative exit interviews. PA is measured using step count data. Adherence data, including daily self-monitoring of blood glucose and medication adherence, are collected through the ABMS IVR system.

In addition to recruitment, retention, and adherence-related measures, other feasibility measures include process, resource, and management measures such as attrition rate, coach time, time to recruit participants, and technology delivery-related issues (Table 3).

**Table 2:** GODART^a^ outcome measures.

			Instrument	Mode of collection	Collection timepoint	Outcome type
**Feasibility**						
	Recruitment, retention, completion, and adherence		See Table 3 for more details	Study logs	Ongoing	Primary
**Clinical**						
	**Blood sample**					
		HbA_1c_^b^	Fasting blood draw	Laboratory visit	Baseline and month 6	Secondary
		Lipid profile	Fasting blood draw	Laboratory visit	Baseline and month 6	Exploratory
	**Biometric**					
		Height (in)	Stadiometer	Laboratory visit	Baseline and month 6	Secondary
		Weight (lb)	Weight scale	Laboratory visit	Baseline and month 6	Secondary
		Waist circumference (in)	Measuring tape	Laboratory visit	Baseline and month 6	Secondary
		Blood pressure (mm Hg).	Sphygmomanometer	Laboratory visit	Baseline and month 6	Secondary
**Patient-reported**						
	Distress related to diabetes		Diabetes Distress Scale	Survey	Baseline and month 6	Secondary
	Diabetes management self-efficacy		Diabetes Empowerment Scale	Survey	Baseline and month 6	Exploratory
	Medication adherence		Amount or frequency as prescribed	IVR^c^	Daily	Exploratory
	Self-monitoring of blood glucose		Measured or not	IVR	Daily	Exploratory
	Anxiety and depression		Patient Health Questionnaire-4	Survey	Baseline and month 6	Exploratory
	Health-related quality of life		EQ-5D-5L	Survey	Baseline and month 6	Exploratory
	Step count		Pedometer	IVR	Daily	Exploratory
	Quality, perceived ease of use, and user satisfaction of speech-based interfaces		Speech User Interface Service Quality	Survey	Month 6	Exploratory
	System usability		System Usability Scale	Survey	Month 6	Exploratory
	Acceptability of the intervention		Theoretical framework of acceptability	Survey	Month 6	Exploratory

^a^GODART: Gamified Optimized Diabetes Management with Artificial Intelligence-Powered Rural Telehealth.

^b^HbA_1c_: hemoglobin A_1c_.

^c^IVR: interactive voice response.

**Table 3:** Feasibility outcomes.

Metric and outcome		Outcome variable computation method
**Process**		
	Time taken to recruit	Time from study activation to enrollment of the last participant
	Recruitment rate	Number of participants enrolled per recruitment time period
	Retention rate	Percent of participants completing the trial
	Enrollment rate	Enrolled of eligible or approached
	Decline rate	Percent of eligible individuals who declined participation
	Completion rate	Percent of enrolled participants who completed the study
	Protocol adherence	Total number of sessions attended of the total 24 coaching sessions; number of monitoring calls answered
	Challenges in collecting blood samples using LabCorp	Qualitative data from patients
	Time to complete data collection	Time to complete pre- and postdata collection
	Duration of human coaching	Time to schedule and conduct orientation and diabetes education
	Data entry errors	Data entry errors
	Percent missing data	Percent missing data
**Resource**		
	Patients’ technical difficulties with technology	Number of issues experienced by participants in technology, glucometer, and LabCorp visits
	Number of replacement glucometers requested	Total cost and number of items purchased
	Number of participant contacts by the team required to complete survey data	Number of contacts
**Management**		
	Fidelity across conditions	Number of deviations from protocol per week of technical issues in interactive voice response
	Fidelity across conditions	Frequency, duration, and nonbusiness hour times requested of contact or support

### Data Management

Data are being collected and managed in a custom-designed REDCap database. Data will be exported to the statistical analysis software, using relational logic checks to identify internal inconsistencies and out-of-range values. Systematic cleaning and verification of all collected data will be conducted to examine ranges, provenance, and missing values. Any inaccurate data points, outliers, and influential cases will be identified and corrected within REDCap. Dashboards and reports will be created and periodically updated to assist the team with recruitment and retention efforts, and track and minimize missing data.

### Sample Size Justification

The primary objective of this feasibility study is not to serve as a definitive, well-powered trial, but to conduct a pilot study that is sufficiently large to estimate design parameters, as well as assess feasibility parameters, such as attrition rate, adherence, and pre-post correlation [58-60]. Accounting for the bias and precision of the estimated pooled SD can help us conduct rigorous power and sample size calculations for a subsequent confirmatory study [61]. We are randomizing 88 participants across four conditions (22 per experimental condition). This is almost twice the number of participants per condition recommended when undertaking pilot studies, when there is no prior information on which to base a sample size [62]. The goal of our pilot study is also to estimate precise effect sizes and design parameter estimates, which requires sufficiently large pilot studies. Based on simulations published by Teare et al [61], an external pilot study should have at least 70 participants when estimating the pooled SD for a continuous outcome. Because we are using a factorial experiment, for each of the main effects, we will have 88 participants, with 44 participants in each of the two comparators. Hence, we have a sufficiently large pilot study to detect precise estimates of effect sizes for the two main effects and other design parameter estimates, such as pre-post correlation.

We also present power calculations of our experiment based on our sample size of 88. Assuming a type 1 error rate of 0.05, a conservative pre-post correlation of 0.5 in ANCOVA, and an attrition rate of 15%, we will have 80% power to detect an effect size (Cohen *d*) of 0.572 in our primary outcome. This would imply a main effect difference of 0.86% units of HbA_1c_, assuming an SD of 1.5% [63]. Even if we assume the pre-post correlation is 0.3, which is very conservative, we will have 80% power to detect an effect size of 0.63 for the main effect. Assuming a more realistic pre-post correlation of 0.7, we can detect an effect size of 0.472. Missing outcomes data will be imputed using maximum likelihood estimation or a multiple imputation procedure, and primary analyses will not be restricted to only completers. Hence, our proposed study sample size of 88 participants is powered to detect an even smaller effect size than suggested here since the effective sample size, even assuming an attrition of 15%, would be between 74 and 88. Thus, our pilot study is well-powered to detect moderate to medium effect sizes. These power calculations were done using the MOST package in R (R Core Team), developed by Dziak et al [64].

### Statistical Analyses

#### Feasibility-Related Analyses

Descriptive statistics of feasibility measures such as adherence, dropouts, and recruitment will be estimated and provided. Bias-corrected bootstrap CIs of the Spearman correlation between baseline and 6-month assessment will be reported. For participants identified through the i2b2 system at UAB Health System, we are tracking the following: (1) no response, (2) screened but failed, (3) screened as eligible but did not consent, (4) screened as eligible and consented, (5) enrolled but withdrew before the intervention started, (6) started the intervention but withdrew during the study, and (7) completed the intervention and final data collection. In addition to the descriptive measures of recruitment outcomes, we will run at least a binary logistic regression model to explore and predict factors associated with successful recruitment outcomes. Successful recruitment will be defined as participants screened as eligible, consented to participate, completed the intervention, and completed the final data collection. Age, gender, race, location status (urban vs nonurban), and health insurance status will be covariates.

#### Analytic Strategy for Preliminary Effectiveness Analyses

We will also estimate effect sizes as preliminary effectiveness estimates. All statistical analyses will follow an intent-to-treat approach. In line with a pilot study, we will also report 75% and 85% CI estimates in addition to the traditional 95% CIs to further aid in the statistical interpretation of the findings [65]. We will use linear mixed models to estimate effects by accounting for correlated data and benefit from the maximum likelihood estimation procedure that uses all information in the dataset in the presence of missing outcome data. Our overall approach will be similar to Pellegrini et al [38]. To evaluate the main effect of each component on pre-post differences in outcomes, we will use baseline as the reference cell to determine differences in change across time (ie, 6 months vs baseline) [38]. However, these effects will be statistically modeled as component-by-time interactions, with the 6-month outcome as the primary end point. We will also include two-way interactions between components (eg, Factor 1 by Factor 2 by time interaction). We will then identify all the active components or combinations of components, where active is operationalized as a mean reduction of 0.5 units (%) in HbA_1c_ between baseline and month 6 [66,67]. Similar analyses will be done for secondary outcomes. Where information related to minimally clinically important differences is available, we will use it to assess whether the combination is active. For example, an average 5% weight loss is considered to be active in the case of weight. We will leverage the generalized linear modeling framework and resampling-based approaches for nonnormal outcomes. We will conduct exploratory analyses to assess biases in the self-reported PROs. While we are not statistically powered to do mediation or moderation analyses, in line with the MOST Preparatory Phase, we will conduct exploratory mediation and moderator analyses using our tracking data as potential mediators or moderators.

#### Analysis of Qualitative Interviews

Collected interview data will be analyzed using a phenomenological approach. Two researchers will independently code the transcribed audio-recorded interviews. They will meet after coding every four transcripts to discuss and resolve any discrepancies in coding. A third researcher will be available to adjudicate unresolved discrepancies. Coding will follow an inductive, emergent approach grounded in the transcript data, using both descriptive and in vivo codes. The coding process will involve two levels: first-cycle coding, where initial codes are assigned using in vivo coding, and second-cycle coding, where these codes are refined and grouped into broader themes using pattern coding [68]. The codebook structure will include themes, codes, and text examples of quotes. Thematic saturation will guide the final sample size. We will enhance trustworthiness through triangulation, peer debriefing, and maintaining an audit trail. All qualitative analyses will be done using NVivo 12 Plus (Lumivero).

## Results

We conducted 1938 phone contact attempts, resulting in 1192 participants completing phone screening. Of those, 162 were deemed potentially eligible and consented to screen for HbA_1c_. As of October 2025, 88 participants who were randomized and enrolled in the study have completed the 6-month follow-up. We anticipate completing data analysis by December 2025. Table 4 outlines the study timeline and anticipated completion of key milestones.

**Table 4:** Timeline of study progress and anticipated target completion.

	2022					2023					2024					2025			
	Q1	Q2	Q3	Q4	Q1		Q2	Q3	Q4	Q1		Q2	Q3	Q4	Q1		Q2	Q3	Q4
Institutional Review Board approval				✓	✓														
Manual of Operating Procedures development and refinement	✓	✓	✓	✓	✓		✓	✓											
Recruitment period									✓	✓		✓	✓	✓					
Intervention period									✓	✓		✓	✓	✓	✓		✓		
Baseline assessment									✓	✓		✓	✓	✓					
6-month assessments									✓	✓		✓	✓	✓	✓		✓		
Data management and quality control									✓	✓		✓	✓	✓	✓		✓	✓	✓
Exit interview									✓	✓		✓	✓	✓	✓		✓		
Data analysis															✓		✓	✓	✓
Publications																	✓		✓

## Discussion

### Principal Findings

This paper sought to outline the study protocol used in a pilot study grounded in SCT, aiming to assess the feasibility of an AI-assisted, low-cost, lifestyle modification intervention for glycemic control in the Deep South. The intervention is designed to be accessible through a standard telephone service and leverages MOST, an efficient and rigorous resource-management and continuous-improvement framework. The MOST design allows us to explore (1) a fixed versus adaptive (gamified) rewards program, and (2) automated versus human-delivered weekly health coaching for four total experimental conditions to see which is most effective for T2DM management. Findings from this pilot and feasibility study will inform the resources needed for a well-powered optimization trial. We will estimate key design parameters—such as effect sizes and pre-post correlations—to guide future sample size calculations. The study also helps identify protocol modifications to improve scalability, recruitment, and participant-centeredness.

The rationale for this study comes from the rising prevalence of T2DM in the Deep South and the lack of self-management in rural, underserved, or low digital literacy populations [69]. Given this escalating prevalence of T2DM, challenges in access to primary care and time available to assist patients in self-management of T2DM, coupled with disparities in its management, there is a need for an optimized, low-cost, scalable intervention that complements primary care for T2DM management to ensure health equity. Synchronous human coaching has been shown to be effective in glycemic control for patients with T2DM [70] and has been explored in rural-specific settings as well [71,72]. A nurse-led telecoaching study for rural patients was successful and cost-effective in T2DM management [73-80]. However, providing 10-15-minute live telephone calls to each patient at frequent intervals presents a significant barrier for scalability. This led to the development of automated health coaching delivered via an automated phone call by an IVR system. Participants use the keypad of any phone (landline, mobile, or smartphone) to interact with the IVR system, where the human coach is replaced by high-quality recorded interactive scripts [81]. IVR-delivered coaching is thus far more scalable than traditional delivery. IVR-based behavior monitoring has been shown to be effective in driving behavior changes [81]. Hence, the proposed intervention could contribute to an interprofessional primary care system, alleviating access issues. Our proposed resource optimization experimentation arms of fixed and adaptive gamified rewards are relevant in the context of existing wellness programs offered by employers and payors to incentivize behavior change. The intent is to promote behavior tracking that can facilitate behavior change where needed, leading to improved health outcomes and improved employee productivity, and less health care expenditure, such as emergency department visits or avoidable hospitalizations. The cost-effectiveness sustainability was not part of our pilot study; this is an important but different aspect of the Multiphase Optimization Strategy Preparatory phase and will be addressed in our future work using approaches such as microsimulations [82]. The GODART project aims to take a step toward building such a system. An overview of the innovative aspects and limitations is discussed below.

### Strengths and Limitations

Our study has multiple strengths and innovative methodologies. From a technological standpoint, we combine NLP with standard telephone-delivered monitoring and coaching. By incorporating natural language understanding (AI) techniques, we aim to minimize the user burden in self-monitoring, which requires minimal time and low health literacy levels [83,84]. The study uses AI to allow participants to “speak” their daily updates into the phone for recording, significantly easing the data collection process. Additionally, we are implementing a gamified rewards scheme based on data collection done via phone to improve self-efficacy and self-monitoring behavior [31]. Gamification is widely recognized for its potential in managing chronic diseases, and our study leverages this approach through a simple reward system [85].

From the study design perspective, an additional strength is the adoption of the MOST framework, which emphasizes efficient resource management and continuous improvement [36]. Our study objectives align with MOST’s preparatory phase, and it is also structured to serve as the preparatory phase for a future large-scale MOST optimization phase. MOST’s preparatory and optimization phases are more efficient for intervention development than a traditional randomized controlled trial (RCT) since it would take substantially greater resources (time and sample size per RCT) to meet this goal [37]. These MOST phases in our study will provide more information than a typical feasibility and well-conducted pilot study using an RCT approach. Further, in MOST, intervention optimization could be an iterative and continuous process compared to a traditional RCT.

From the data collection and engagement perspective, our study leverages simple telehealth technology based on IVR to collect periodic data from participants and keep them engaged throughout the study period. Collecting these data as part of the diabetes self-management activities instead of apps or logs is meant to reduce the participant burden and keep the data collection process simple and accessible, regardless of patients’ health literacy or technological access. Further, analyses of these data can help develop learning models that can enable further optimizations through the development of just-in-time adaptive interventions [86,87]. Finally, blood draws are collected at the LabCorp location nearest to each patient’s home. This strategy minimizes potential delays by reducing the need for patients to travel to specific clinic locations.

There were several limitations to this study. First, the study design required participants to engage daily in a phone-based intervention, which may limit generalizability, as individuals who enrolled were likely more motivated at baseline than the broader population of adults with type 2 diabetes. As a result, the findings may be most applicable to patients already inclined to engage in self-management, highlighting the need for future research to develop strategies that also reach individuals with lower baseline motivation.

Second, although our system uses AI to process information collected through IVR calls, the coaching recommendations themselves are not generated by AI. Instead, they are developed by a human coach and then delivered asynchronously via IVR. Thus, the automated call arm still depends on human input and is not entirely independent of human resources. Since the design of this study, there has been a major shift in AI technology with the availability and mainstreaming of large language models. Future iterations of this project will be able to leverage LLM-based approaches to further enhance AI-based ABMS and AI-based health coaching.

We also asked participants to report whether they were checking their blood sugar, but did not collect actual glucose values. This was due to the lack of a clinical remote monitoring team available to respond to hypo or hyperglycemia alerts. Future studies may incorporate continuous glucose monitoring or partner with remote monitoring teams, such as registered nurses within a health care system, to respond to critical alerts and provide timely feedback and support.

Additional limitations include the exclusion of non-English speakers, which may reduce the generalizability of findings to linguistically diverse populations. All behavioral data was self-reported, which introduces the potential for recall bias—particularly underreporting—since more detailed entries (eg, food logs) led to longer IVR calls and may have discouraged full reporting. Additionally, the study does not include a scale to assess social desirability bias in the self-reported PROs, which we will incorporate in our future study. Coaching fidelity was not comprehensively evaluated: the automated coaching was still guided by a human coach, and the degree of standardization or comparability between the human and automated arms was not assessed. Given the applications of LLMs, we anticipate this issue to be addressed by the adoption of LLM in our GODART technology platform.

### Conclusions

The rising prevalence of T2DM, specifically in the Deep South and rural areas, calls for interventions that can be delivered remotely yet with minimal digital complexity. These could serve as scalable digital care management plans offered in primary care settings to address the current primary care limitations due to limited access and time. This study seeks to test the feasibility of a telehealth and AI-driven intervention designed to be easily accessible, even for individuals with minimal digital literacy. By gathering data from this study, we intend to lay the groundwork for a future large-scale optimization trial as a step toward building an interdisciplinary primary care system that could integrate health technology effectively, ensuring that patients receive care at the right time and place.

## Appendix

Multimedia Appendix 1 Branching logic for ABMS and coaching calls.

Multimedia Appendix 2 Qualitative interview guide.

## Abbreviations

ABMS: automated behavior monitoring system
AI: artificial intelligence
API: application programming interface
GODART: Gamified Optimized Diabetes Management with Artificial Intelligence-Powered Rural Telehealth
HbA_1c_: hemoglobin A_1c_
IVR: interactive voice response
MOST: Multiphase Optimization Strategy
NLP: natural language processing
PA: physical activity
PRO: patient-reported outcome
RCT: randomized controlled trial
REDCap: Research Electronic Data Capture
SCT: social cognitive theory
SKILLD: Spoken Knowledge in Low Literacy in Diabetes Scale
T2DM: type 2 diabetes mellitus
UAB: University of Alabama at Birmingham

## Data Availability

The datasets used or analyzed during the current study are available from the corresponding author upon reasonable request. The trial data will be publicly available for publication in international peer-reviewed journals, regardless of whether the results are positive or negative.
