# Supplementary material for: Gamified Optimized Diabetes Management With Artificial Intelligence–Powered Rural Telehealth Intervention (GODART): Protocol for an Optimization Pilot and Feasibility Trial
Source: JMIR Res Protoc. 2025 Dec 5;14:e70271. doi: 10.2196/70271 (PMC12717512; doi:10.2196/70271)
Supplement: Multimedia Appendix 1 [file resprot_v14i1e70271_app1.pdf]

Interactive Voice Response system TRACKING AND GOAL SETTING call script

Daily Tracking Calls

Introduction					
ITEM	Initiating Prompt	Participant Action	Follow-Up Prompt	Participant Action	System Action
PIN	*[Welcome message at random from Greetings Bank depending on if call is caller initiated or IVR-initiated]*	[unique 4 digit pin]	Welcome back! [*New Content Prompt weeks 2-4 of each month]*		

Daily Physical Activity Questions					
ITEM	Initiating Prompt	Participant Action	Follow-Up Prompt	Participant Action	System Action
Physical Symptom	Have you experienced any physical symptoms or complaints that could interfere with your physical activity?<br><br>You can Press 1 for Yes, or<br>Press 2 for No.	Yes or 1<br>No or 2	[If yes]<br>Please stop exercising and contact your doctor to discuss these health issues further. You will receive a telephone call from the study staff to follow up on this matter within the next 48 hours.		
Wear Pedometer	Did you wear your Fitbit or pedometer today?	Yes or 1<br>No or 2			[If No]<br><br>Proceed to **Physical Activity**

	You can Press 1 for Yes, or<br>Press 2 for No.				[If Yes]<br>Continue with Step Questions (**How Many Steps**)
How Many Steps	How many steps did you take today?	0-20,000	Thank you for entering your steps for today.		Proceed to **Medication Adherence Questions**
Physical Activity	Did you participate in any moderate intensity or greater physical activity today?<br><br>You can Press 1 for Yes, or<br>Press 2 for No.	Yes or 1<br>No or 2	Thank you for entering your physical activity.		Proceed to **Yes Physical Activity or Medication Adherence**
Yes- Physical Activity	Approximately how many minutes did you spend participating in moderate intensity or greater physical activity throughout the day today?				

Daily Medication Adherence Questions					
ITEM	Initiating Prompt	Participant Action	Follow-Up Prompt	Participant Action	System Action
Medication Adherence	Did you take your diabetes medications today?<br><br>You can Press 1 for Yes, or Press 2 for No.	Yes or 1<br>No or 2			[if yes]<br>Proceed to **Medication Dose**<br><br>[if no]<br>Proceed to **Blood Sugar Monitoring** Questions
Medication Dose	Did you take your diabetes medications in the amount prescribed by your doctor?<br><br>You can Press 1 for Yes, or Press 2 for No.	1=Yes<br>2=No			Proceed to **Medication Time**
Medication Time	Did you take your diabetes medications at the appropriate time today?<br><br>You can Press 1 for Yes, or Press 2 for No.	Yes or 1<br>No or 2	Thank you for entering your medication information today.		Proceed to **Blood Sugar Monitoring** Questions

Daily Blood Sugar Monitoring Questions					
ITEM	Initiating Prompt	Participant Action	Follow-Up Prompt	Participant Action	System Action
Blood Sugar Log	Did you test your blood sugar in the morning today?<br><br>You can Press 1 for Yes, or<br>Press 2 for No.	Yes or 1<br>No or 2	Thank you for entering your blood sugar monitoring information today.<br><br>Remember that diet, exercise, medications, and blood sugar logging are each important for taking care of diabetes.		

Daily Diet Questions					
ITEM	Initiating Prompt	Participant Action	Follow-Up Prompt	Participant Action	System Action
Initial instruction about food log	We would like to know about your food intake today. Be sure to include the amount of each food or drink item that you consumed throughout the day.				
Diet Log 1	Did you have breakfast today?<br><br>You can Press 1 for Yes, or<br>Press 2 for No.	Yes or 1<br>No or 2	[if no] proceed to Diet Log 2		**Repeat** as needed.

	What did you eat or drink for breakfast?	States first food/drink items.			
	Thanks, did you eat anything else for breakfast?<br><br>You can Press 1 for Yes, or<br>Press 2 for No.	Yes or 1<br>No or 2	[if yes]<br>Okay, what other food or drink items did you have today for breakfast.		**Repeat** as needed.
Diet Log 2	Did you have lunch today?<br><br>You can Press 1 for Yes, or<br>Press 2 for No.	Yes or 1<br>No or 2	[if no] proceed to Diet Log 3		**Repeat** as needed.
	What did you eat or drink for lunch?	States food/drink items.			

	Thanks, did you eat anything else for lunch?<br><br>You can Press 1 for Yes, or<br>Press 2 for No.	Yes or 1<br>No or 2	[if yes]<br>Okay, what other food or drink items did you have today for lunch.		**Repeat** as needed.
Diet Log 3	Did you have dinner today?<br><br>You can Press 1 for Yes, or<br>Press 2 for No.	Yes or 1<br>No or 2	[if no] proceed to Diet Log Final		**Repeat** as needed.
	What did you eat or drink for dinner?	States food/drink items.			
	Thanks, did you eat anything else for dinner?<br><br>You can Press 1 for Yes, or<br>Press 2 for No.	Yes or 1<br>No or 2	[if yes]<br>Okay, what other food or drink items did you have today for dinner.		**Repeat** as needed.

Diet Log Final	Was there anything else you had today? This could be a snack or a drink.<br><br><u>You can Press 1 for Yes, or<br>Press 2 for No.</u>	Yes or 1<br>No or 2	[if yes]<br>Okay, what was it?		**Repeat** as needed until No
	Thanks, did you have any other snacks or drink?<br><br><u>You can Press 1 for Yes, or<br>Press 2 for No.</u>	Yes or 1<br>No or 2	[if yes]<br>Okay, what was it?		**Repeat** as needed until No
	Thank you for entering your food record data.				

WEEKLY Goal Setting/Coaching Calls

Introduction					
ITEM	Initiating Prompt	Participant Action	Follow-Up Prompt	Participant Action	System Action
PIN	*[Welcome message at random from Greetings Bank depending on if call is caller initiated or IVR-initiated]*	[unique 4 digit pin]	Welcome back!<br>[*New Content Prompt weeks 2-4 of each month]*		

Goal Setting (Diet)					
ITEM	Initiating Prompt	Participant Action	Follow-Up Prompt	Participant Action	System Action
Meal Frequency	One of our goals is to space meals evenly throughout the day. How often have you been skipping meals or going longer than 4 hours between eating occasions?<br><br>You can Press 1 for Often, or Press 2 for Sometimes, or Press 3 for Never.	Often<br>Sometimes<br>Never			If participant selects **sometimes** or **never** AND there is prior meal timing data, proceed to **Yes Meal Frequency**<br><br>If participant selects **often**, proceed to **No Meal Frequency**

Yes Meal Frequency	That's great. You are doing a wonderful thing for your health. Eating consistently throughout the day helps keep blood sugar stable and provides energy throughout the day.				Proceed to **Total Carbohydrate**

No Meal Frequency	We would like to know what got in the way of you eating at regular intervals this week.<br><br><u>Press 1 for Not enough time to eat or too busy</u><br><u>Press 2 for Not hungry</u><br><u>Press 3 for Trying to lose weight</u><br><u>Press 4 for Stress</u>	1=Not enough time to eat or too busy<br>2=Not hungry<br>3=Trying to lose weight<br>4=Stress			Sorry things got in the way this week.<br><br>If 1:<br>It can be helpful to set reminders on your phone or watch every 4 hours to ensure you make time for meals or snacks.<br><br>If 2: If you don't have an appetite, it is still helpful to eat even a small snack to manage blood sugar. Aim to eat at least 3 times per day in some combination of meals or snacks.<br><br>If 3: Skipping meals may seem like a fast track to lose weight, but it prevents you from achieving optimal blood sugar control and may actually make weight loss more difficult. Not eating regularly can cause blood sugar to drop too low and hunger to increase to a point

					where you overeat at the next meal or snack.<br><br>If 4: Stress can negatively impact your physical health in several ways. We recommend reaching out to your doctor for behavioral health resources to help manage stressful events or circumstances in your life.<br><br>Let’s focus on eating every 3-4 hours this week. This will help make sure that your blood sugar, energy, and hunger levels steady throughout the day.<br><br>Proceed to **Total Carbohydrate High or**<br>**Total Carbohydrate Met or**<br>**Total Carbohydrate Low**

Total Carbohydrate Goal (1st Week)	Let's set a goal this week to focus on carbohydrate intake. Remember that carbohydrates have the biggest impact on your blood sugar. Aim to get about half of your energy from carbohydrate foods (grains, fruits, vegetables, and milk).				
Total Carbohydrate High	[If system calculates carb intake > 60% of total kcal]<br><br>Based on your food record data from the past week, it seems like your overall carbohydrate intake was a little higher than our goal.<br><br>[A human health coach will look at the data to determine the best target for goal setting. The data entered into the sytem will be based				Proceed to **Total Carbohydrate High Goal**

	on starch/added sugar vs. fiber.]<br><br><br>The foods highest in carbohydrates from the past week were:<br>1)___________<br>2)___________<br>3)___________				
Total Carbohydrate High Goal	Let's set a goal this week to focus on carbohydrate intake.<br>Reduce______ or Replace ______with _____.<br>Remember that carbohydrates are what has the biggest impact on your blood sugar. Carbohydrates from whole grains and vegetables are the best choice because they are high in fiber, which helps with blood sugar control and keep you feeling full. Aim to get about half of your energy from				

	carbohydrate foods (grains, fruits, vegetables, and milk).				
Total Carbohydrate Met	[If system calculates carb intake 40-60% of total kcal]<br><br>Based on your food record data from the past week, your overall carbohydrate intake is in an appropriate range. This is excellent news, great job! Aim to get about half of your energy from carbohydrate foods (grains, fruits, vegetables, and milk).				
Total Carbohydrate Low	Based on your food record data from the past week, your overall carbohydrate intake may be too low.				Proceed to **Total Carbohydrate Low Goal**

Total Carbohydrate Low Goal	Let's set a goal this week to focus on carbohydrate intake.<br>Increase ______ or Replace ______ with ________.<br>Although carbohydrates raise blood sugar, they are still an important part of our diet. Taking in too little carbohydrate may make blood sugar dip too low and/or limit vital nutrients in our diets. Aim to get about half of your energy from carbohydrate foods (grains, fruits, vegetables, and milk).				Proceed to **Carb Distribution Met** or **Carb Distribution Unmet**
Carbohydrate Distribution Met	Based on your food record data from the past week, you have been eating similar amounts of carbohydrate at each meal and snack. Great work! This will help keep blood sugar and				Proceed to **Saturated Fat High** or **Saturated Fat Met**

	energy levels stable throughout the day.				
Carbohydrate Distribution Unmet	Based on your food record data from the past week, your carbohydrate intake may be inconsistent throughout the day.				Proceed to **Carbohydrate Distribution Unmet Goal**
Carbohydrate Distribution Unmet Goal	Let's set a goal this week to focus on including moderate amounts of carbohydrate at every meal and snack. One-quarter of your plate at meals can be occupied by high-carb foods. Remember that carbohydrates can be found in the highest amounts in starches/grains, dairy foods, and fruits.				Proceed to **Saturated Fat High** or **Saturated Fat Met**
Saturated Fat Goal (1st Week)	Let's set a goal this week to focus on saturated fat intake.				

	Remember that Saturated fat is the kind of fat that can damage heart health and should be limited in the food we eat. For this first week, let's make it a goal to eat only lean proteins like chicken, turkey, fish, and beans and replace any full-fat milk and cheese with lower-fat alternatives.				
Saturated Fat High	[If system calculates sat fat intake > 10% of total kcal.]<br><br>Based on your food record data from the past week, it seems like your overall saturated fat intake was a little higher than our goal.<br><br>[A human health coach will look at the data to determine the best target for goal setting.]				Proceed to **Saturated Fat High Goal**

	The foods highest in saturated fat from the past week were:<br>1)___________<br>2)___________<br>3)___________				
Saturated Fat High Goal	Let’s set a goal this week to focus on saturated intake. Reduce_____ or Replace ______ with ________. Saturated fat is the kind of fat that can damage heart health and should be limited in the food we eat. This kind of fat is found in fatty cuts of meat, full-fat dairy, and some fried foods. Aim to eat more healthy fats that improve heart health, such as nuts and olive oil.				

Saturated Fat Met	[If system calculates sat fat intake < 10% of total kcal.]<br><br>Based on your food record data from the past week, your overall saturated fat intake is in an appropriate range. Great work! Continue focusing on foods low in saturated fat every day.				Proceed to **Physical Activity** Questions
Diet Barriers	We want to learn a little bit more about how these goals are working for you and your life right now Did any of the following prevent you from meeting your nutrition goals this week?		Eating problems associated with strong emotions (e.g., stressed, anxious, depressed, angry, or bored). (1)<br><br>Eating problems because of hunger or food cravings. (2)<br><br>Eating problems because family or friends are not supportive of		If 1: It is very common for emotions to prevent people from reaching their nutrition goals. Try to identify patterns in your eating behavior that might be tied to your emotions. Being aware of these patterns can help you change them to be more supportive of a healthy diet. If you feel you need to talk with someone about what you’re

			your efforts to eat healthy foods. (3)<br><br>Eating problems because you are away from home (e.g., fast food, restaurants, traveling, or at social events). (4)<br><br>Eating problems because you feel deprived of foods you enjoy (5).<br><br>Eating problems because you feel discouraged due to lack of results. (6)<br><br>Eating problems because you are too busy with family, work, or other responsibilities. (7)<br><br>None of the above (8).		feeling, your primary care physician can connect you with a mental health professional.<br><br>If 2: If you are hungry, then your body is telling you it needs fuel! Adding plenty of vegetables to your meals is a great way to fill up your stomach without adding too many carbohydrates or kcalories. Moderate portions of lean protein or healthy fats are also excellent ways to make you feel full. As for cravings, the best way to beat them is to allow yourself a small portion once every 1-2 weeks. A healthy diet is all about balance, and that includes a small treat occasionally. The key is portion control.<br><br>If 3: I am sorry that you have faced a

					lack of support from friends and family; that can make healthy eating challenging. If at all possible, try to include them in your health journey by explaining your goals, or inviting them to cook or enjoy a healthy meal with you.<br><br>If 4: It is still possible to eat well while away from home, but it may require a bit more planning. When eating out, take a look at restaurant options and their menus ahead of time; look for places that offer fruits and vegetables and that use healthy food preparation techniques (i.e, grilling or roasting instead of frying). At social events, do your best to choose healthy foods, but if they are not available, the key is

					to keep your portions small with foods that are high in carbohydrates, sugar, and/or saturated fat.<br><br>If 5: For cravings, the best way to beat them is to allow yourself a small portion once every 1-2 weeks. A healthy diet is all about balance, and that includes a small treat occasionally. The key is portion control.<br><br>If 6: It can be frustrating to feel like you are making big changes to your diet and not see the results you want. Try to be patient and remember that it can take time for your body to catch up with all the changes you're making to your diet. Good health takes time to achieve, and consistency pays off.

					If 7: A busy lifestyle can make it difficult to implement big changes in your eating habits. It’s important to keep your health as a top priority, however. Setting aside a small amount of time each week to have a very general plan of your meals and snacks can make a big difference.

Goal Setting (Physical Activity)					
ITEM	Initiating Prompt	Participant Action	Follow-Up Prompt	Participant Action	System Action
Step Goal (1st Week)	Let’s set this week’s step goal. Your initial goal is to take 3000 steps per day this week.				
Pedometer Yes	[If pedometer was worn 7d/wk.]<br><br>You reported that you wore your pedometer every day last week. We’re so glad you have gotten in the				

	habit of wearing the pedometer daily. This will help you track exercise and help you stay active.				
Pedometer No	It looks like you did not wear your pedometer every day last week. Tracking your steps will motivate you to be more physically active. Wear your Fitbit or pedometer as much as possible. If it is lost or not working, call us at…				Proceed to **Step Count**
Step Count	Based on the step counts you provided last week, you averaged _____ steps per day.		[step goal met] Way to go! That's more steps than you reported last week! You are on the right path. Let's try adding another 250 steps to your daily goal. Have fun, but always keep your health and safety in mind		Proceed to **Medication Adherence** Questions

			[Same as last data point; under goal]<br>That's the same number of steps you reported last time. Beat your record by taking more steps this week. Remember we're aiming to add 250 steps to your daily step count.<br><br>[Less than last time]<br>That's fewer steps than you reported last time. Focus on being more active this week. You can do it.		
Step Count Barriers	[Same as OR less than last time. Still under 10k]<br>We would like to know what prevented you from increasing your steps this week. Did any of the following make it difficult for you to exercise this week?		Feeling stressed, anxious, depressed, angry, or bored (1)<br><br>Exercise causes pain and discomfort for me (2)		If 1: Experiencing strong emotions can make it hard to find the motivation for exercise. However, you might find that engaging in physical activity helps change your mood. It can be hard to get started,

			Family or friends are not very supportive. (3)<br><br>I was away from home. (4)<br><br>My daily schedule is different from one day to the next. (5)<br><br>Feel discouraged due to lack of results. (6)<br><br>Being too busy with family, work, or other responsibilities. (7)<br><br>None of the above (8)		but you can begin with just 5 minutes and see how you feel. You might find that you feel better and more motivated to keep going!<br><br>If 2: We certainly don’t want you to engage in activities that cause pain. Switching from a high-impact activity (jogging, aerobics, some sports) to a low-impact activity (swimming, cycling) can help with some causes of pain or discomfort. It might be worth asking your doctor or requesting a referral to help manage pain associated with physical activity.<br><br>If 3: Pursuing your health goals without a support system can be difficult. It might be

					beneficial to have a conversation with friends or family about your health goals and why you've set them. You can also invite them to be active with you; having a workout buddy makes it easier and more enjoyable to be active!<br><br>If 4: The nice thing about counting steps is that you can do it anywhere! Even if you are away from home, you can boost your step count with simple changes, such as taking the stairs for a few flights, or parking further from your destination. Being away from home does not have to mean that your physical activity stops.<br><br>If 5: Maintaining habits can be

					challenging when your schedule is variable. However, it is possible with some planning. It can be useful to set aside a few minutes each morning to look at your schedule and identify pockets of time for a quick walk. Treat exercise like any other appointment- add it to your calendar, and make it non-negotiable!<br><br>If 6: Most health habits require consistency over the long-term for the most benefit, and exercise is no exception. Moving you body more over the course of months (not days or weeks) will have a positive impact on your blood sugar control and weight

					management. Consistency is key!<br><br>If 7: Adding exercise into an already busy schedule can be daunting, but it is so important. Make your health a priority as often as possible. Exercise is one way of keeping yourself well so that you can carry out all the responsibilities in your life.

Goal Setting (Medication Adherence)					
ITEM	Initiating Prompt	Participant Action	Follow-Up Prompt	Participant Action	System Action
Medication Goal (1st Week)	Being consistent with medications is essential for taking care of your health. Let's				Proceed to **Blood Sugar Goal**

	set a goal of taking your diabetes medications every day this week.				
Medication Adherence Yes	[If took medicines 7d/wk]<br><br>You reported that you took your diabetes medications every day last week. Great work! Being consistent with medications is essential for taking care of your health.				Proceed to **Medication Adherence No**
Med Adherence No	[If medications taken < 7d/wk]<br><br>It looks like you missed taking some medications last week.<br>We would like to know what got in the way of you taking your diabetes		Feeling stressed, anxious, depressed, angry, or bored. (1)<br><br>The medicine has unpleasant side effects. (2)		If 1: Strong emotions can certainly make it more difficult to maintain health habits. Try to identify patterns with missing medication doses and the way you're feeling, perhaps with a log book or an app on your smartphone. Being aware of these patterns is key to changing them

	medications this week. Did any of the following make it difficult for you to take your medications?		Family or friends are not very supportive (3)<br><br>I was away from home. (4)<br><br>My daily schedule is different from one day to the next (5)<br><br>Feel discouraged due to lack of results. (6)		for your health in the future. If you feel you need to talk with someone about what you're feeling, your primary care physician can connect you with a mental health professional.<br><br>If 2: If you notice unpleasant changes in how you feel after taking a prescribed medication, we recommend that you talk with your doctor about these symptoms to see if there's a solution to avoid side effects.<br><br>If 3: Not having a support network can make it more challenging to reach your health goals. If at all possible, I encourage you to talk with friends and family about your goals and why you've set them. Sometimes open communication about the issue can help resolve conflicts.

					If 4: Consider taking your medications like an appointment; you need to plan for them ahead of time. That might mean carrying a small number of pills in your purse or car, and putting a reminder in your calendar or phone so you remember to stop for your dosage while away from your normal routine. This will help with blood sugar control.<br><br>If 5: If your schedule varies throughout the week, set aside some time to “pencil in” when you can take your medications each day, either on a calendar or on your phone. Taking your medications on time will help avoid large swings in blood sugar throughout the day.<br><br>If 6: Patience in seeing results is certainly challenging. Try to remember that very few

					medications exert their effects instantaneously; being consistent with taking your medications over time is likely to yield the best results. However, if you feel that you are not seeing benefit from taking your medications as prescribed for a long time, you might discuss alternative options with your doctor.<br><br>If 7: Being busy certainly makes it difficult to implement lifestyle changes, including taking medications. Maintaining your health needs to be a top priority, however, because everything else (family, work, etc.) depend on you being well. Make the time for your medications so you can feel your best and take care of all your responsibilities.<br><br>If 8: If cost is an issue, definitely talk to your healthcare team about

					the best ways to afford the medications you need. They may be able to connect you with a discount or coupon for your existing medication or switch you to a lower-cost alternative.<br><br>Proceed to **Medication Adherence No Goal**
Medicine Dosage No	You reported that you took a different amount of your diabetes medications than prescribed. Did you most often take more or less than what was prescribed?	1=More<br>2=Less	We would like to know why you altered the dose of your diabetes medications. Please select from the following:	I feel better taking a different amount of the medication. (1)<br><br>I am trying to make my diabetes medications last longer by taking a smaller dose. (2)<br><br>I want to reduce the risk of side effects by taking less medicine. (3)<br><br>Something else (4)	If 1: It can be dangerous to take a different dose (either more or less) than what was recommended by your doctor. If you feel that a different amount would help you feel better, discuss this with your doctor. It may be possible to make changes to your prescription, but always involve your healthcare team before taking a different amount.<br><br>If 2: Taking too little medication can put your health at risk. It's important to have an honest talk with your healthcare team about

					medication costs. They may be able to provide a discount or coupon or prescribe a lower-cost alternative.<br><br>If 3: If you notice unpleasant changes in how you feel after taking a prescribed medication, we recommend that you talk with your doctor about these symptoms to see if there's a solution to avoid side effects.
Medication Time No	You reported that you did not take all your diabetes medications at the appropriate time last week. We would like to know what got in the way of taking your medications on time this week.		My schedule kept me from taking medications on time. (1)<br><br>I feel better taking my medications at a different time. (2)<br><br>Something else. (3)		If 1: Taking your diabetes medications on time ensures that you'll feel well throughout the day. Prioritize taking your medications as prescribed to avoid hyperglycemic or hypoglycemic episodes.<br><br>If 2: We recommend talking to your healthcare team before making any changes to how or when you take your medications.

Medication Adherence No Goal	Being consistent with medications is essential for taking care of your health. Let's set a goal of taking your diabetes medications every day next week, in the amount prescribed, and at the right time. This will help you feel your best and keep your blood sugar closer to your goal and keep it as stable as possible.				Proceed to **Blood Sugar Monitoring Questions**

Goal Setting (Blood Sugar Monitoring)					
ITEM	Initiating Prompt	Participant Action	Follow-Up Prompt	Participant Action	System Action
Blood Sugar Goal (1st Week)	Checking your blood sugar every				Proceed to Summary

	day is very important for both you and your healthcare team to monitor the status of your diabetes. Knowing your blood sugar helps you to adjust your diet and your healthcare team to adjust medications if necessary.<br>Let's aim to check blood sugar every day this week.				
Blood Sugar Log Yes	[If blood sugar monitored 7d/wk]<br><br>You checked your blood sugar at least once daily every day last week. Congratulations! You are taking charge of your health and diabetes by monitoring blood sugar on a regular basis. Keep it up!				**Proceed to Summary**

Blood Sugar Log No	[If blood sugar monitored <7d/wk]<br><br>It looks like there were a few days that you missed checking your blood sugar.<br><br>We would like to know what got in the way of you checking your blood sugar this week. Press 1 for Forgot or too busy, Press<br>2 for Ran out of test strips or supplies,<br>Press 3 for Do not like testing blood sugar,<br>Press 4 for Something else		Feeling stressed, anxious, depressed, angry, or bored. (1)<br><br>I hate to stick myself (2)<br><br>Family or friends are not very supportive (3).<br><br>I was away from home. (4)<br><br>My daily schedule is different from one day to the next (5)<br><br>Being too busy with family, work, or other responsibilities (6).<br><br>Feeling discouraged due to lack of results (7)<br><br>The testing supplies are too expensive (8).		If 1: Strong emotions can certainly make it more challenging to maintain habits. It can be helpful to observe your blood sugar logging behaviors in relation to your feelings. Being aware of patterns (for example, you tend to skip checking your blood sugar when you're anxious) is key to modifying the habit. If you feel you need to talk with someone about what you're feeling, your primary care physician can connect you with a mental health professional.<br><br>If 2: Testing blood sugar gets easier with time and practice. Stick with it to get in the habit—it's one of

			None of the above (9).		the most important things you can do to manage diabetes.<br><br>If 3: Pursuing health goals can be challenging without the support of loved ones. It can be helpful to have an honest conversation with friends or family about your health goals and why blood sugar monitoring is important for you.<br><br>If 4: Checking blood sugar away from home might be inconvenient, but it is a vital part of monitoring your diabetes. Keep a small stash of supplies in your purse or car for easy access when on the go.<br><br>If 5 or 6: It can be helpful to set reminders on your

					phone or watch to ensure that you log blood sugar readings. Having information related to blood sugar before and after eating helps your care team modify your diabetes treatment plan so that you can be and feel as healthy as possible.<br><br>If 7: Being aware of your blood sugar patterns plays a big role in maintaining healthy habits. By checking blood sugar regularly, you become even more of an expert in your own body and how it responds to certain foods. As a result, you can make more informed food choices, depending on your blood sugar. Don't underestimate how important this behavior is in

					maintaining your health.<br><br>If 8: If you run out of test strips or supplies, please reach out to the study team at ____________. We can provide these at no cost to you.<br><br>Proceed to **Blood Sugar Log No**
Blood Sugar Log No	Checking your blood sugar every day is very important for both you and your healthcare team to monitor the status of your diabetes. Knowing your blood sugar helps you to adjust your diet and your healthcare team to adjust medications if necessary.<br>Let's aim to check blood sugar every day next week.				**Proceed to Summary**

Goal Setting (Summary)					
ITEM	Initiating Prompt	Participant Action	Follow-Up Prompt	Participant Action	System Action
Summary	Thank you for your continued participation in GODART. You're taking the right steps to manage your diabetes. Let's review the goals we set this week.<br><br>As another measure of progress, here is an update on how much you have earned from completing daily tracking calls. As of today, you have earned ___ dollars. Remember that the more calls you complete, the greater your potential for earning.		The goals we set today are:<br>1)____________<br><br>2)____________<br><br>3)____________		
