# Supplementary material for: Gamified Optimized Diabetes Management With Artificial Intelligence–Powered Rural Telehealth Intervention (GODART): Protocol for an Optimization Pilot and Feasibility Trial
Source: JMIR Res Protoc. 2025 Dec 5;14:e70271. doi: 10.2196/70271 (PMC12717512; doi:10.2196/70271)
Supplement: Multimedia Appendix 2 [file resprot_v14i1e70271_app2.docx]

**Interview Guide**

| **Interviewer Initials:** | **Date:** | **Start Time:** | **End Time:** | **Participant ID:** |
| --- | --- | --- | --- | --- |
|  |  |  |  |  |

**SECTION A: Information about the study**

Hello, and thank you for taking the time to speak with me today. My name is [Name], and I’m a [Study Role] with the Department of Family and Community Medicine. I’d like to learn more about your experiences participating in the GODART type 2 diabetes management study.

Is this still a convenient time to talk about your experiences? **[PAUSE]**

- **If [No]:** No problem. When would be a more convenient time for you to discuss your experiences?
- **If [Yes]:** Thank you for agreeing to participate in this interview. This interview is to understand your experiences with participating in this study.
  - Just a quick reminder: there are no right or wrong answers—we are interested in your honest opinions and experiences.
  - The interview will take about 30 minutes, and you'll receive $25 for your time.
  - Your participation is completely voluntary. You may skip any question or stop the interview at any time.
  - With your permission, we’d like to audio-record the interview. The recording will be stored securely and deleted after we publish the findings.
  - To protect your privacy, all information will be kept confidential, and your name will not be linked to your responses.

Before we start, do you have any questions for me that I can answer? **[PAUSE]**

If you do have questions at any time during the interview or need a break, please do not hesitate to ask. Is it ok if I turn on the recorder now?

I’m turning on the recorder now.

**SECTION B: Participation and expectations**

1. What motivated you to participate in this study?

- - **Probing:** Did your physician or someone else recommend that you join
  - **Probing:** Was there something specific about your diabetes you were hoping to improve?

1. When you first started the diabetes management program, what were you expecting to get out of it?
   - **Probing:** Were you hoping to improve your HbA1c or make changes to how you manage your diabetes day-to-day?
2. Now that you've completed the study, how well do you think it matched up with what you were originally expecting?
   - **Probing:** Can you discuss any specific events that were either pleasantly surprising or disappointing?

**SECTION C: Educational packet**

1. How did you feel about the educational packet you got when the study started?

- **Probing:** Did any part of the packet stand out as especially helpful or unhelpful?
- **Probing:** Did you get a chance to read through the complete educational packet at least once? If yes, was the educational packet easy to follow?

1. In what ways did your conversation with the health coach clarify the information in the Educational Packet?
2. Can you give an example of something the coach helped you understand better?

**SECTION D: Daily monitoring calls**

1. What did you think about the daily monitoring calls you received?
   - **Probing:** Were the calls a reminder to take specific action, like checking your blood sugar or taking your medications on time?
   - **Probing:** Did these calls add any stress to your day?
   - **Probing:** What did you think about the length/duration and content of the call?
2. Were there any challenges or benefits you felt strongly about regarding these daily calls?
   - **Probing:** Can you give a specific example of either a challenge or a benefit?
   - **Probing:** Would you like to change anything about these calls?

**SECTION E: Incentive and duration of calls**

1. Were you aware that you did not receive an incentive on days you missed the calls?
2. Did you find the daily incentives motivating given that you received a fixed amount of incentive each time you answered a call?
3. How do you feel about the incentives and duration of the calls?
   - **Probing:** If you could change one thing about the calls, such as the length or the rewards, what would it be?
   - **Probing:** Were the incentives and duration of the calls in line with what you expected upon enrollment?

### [For Adaptive Rewards Only]

### Were you aware of the reward system where your incentive increased when you answered calls and decreased when you missed them?

### Follow-up: How did you feel about this approach?

### [For Fixed Rewards Only]

### Did you find the daily incentives motivating, knowing that you received a fixed amount each time you answered a call?

### Follow-up: Why or why not?

1. Were you aware that you would not receive an incentive on days you missed the calls?

**SECTION F: Health Coaching**

### [For Participants Receiving Automated Health Coaching Calls]

1. What was your overall experience with the automated health coaching calls?
   - **Probing:** Did you feel that the coaching was personalized to you?
   - **Probing:** Were there any moments where you felt lost or frustrated?
2. How relevant was the content of the automated coaching to your diabetes management?
   - **Probing:** Was the information directly applicable to your situation?
   - **Probing:** Did the weekly coaching calls address all the challenges you were facing?
3. What could be improved about the automated coaching experience?
   - **Probing:** Is there something specific you didn't find useful or thought could be better?
   - **Probing:** Did you ever wish the calls were shorter or longer?
   - **Probing:** Do you think having a human health coach would have been more helpful than the automated health coaching you received?
     1. *If yes:* Can you share how having a human coach might have improved your experience or support?

[For Participants Receiving Human Health Coaching]

1. Can you describe your overall experience with your health coach?
   - **Probing:** Can you give a specific example where the coaching helped you manage your diabetes?
   - **Probing:** Did you feel the coaching was personalized to you?
2. How relevant was the content of the coaching sessions to your own diabetes management?
   - **Probing:** Did the coaching sessions cover topics you felt were missing in your current diabetes care?
   - **Probing:** Were there any topics related to diabetes that were not addressed that you would have liked to discuss?
3. Was there anything about the human coaching that you think could be improved?
4. **Probing:** Was there anything you didn’t find helpful or felt could have been done differently?

**SECTION G: General Questions**

1. Has participating in the study changed how you manage diabetes day to day?
   - **Probing:** Can you share an example of a change you made a plan to continue, if any?
2. Has the study affected your confidence in managing your diabetes?
   - **Probing:** Are there specific aspects where you feel more confident now?
3. Do you have any suggestions or feedback you'd like to share about the study?
   - **Probing:** Was there a moment that stood out to you—either something especially helpful or something you feel needs improvement?
